# *In Vivo* Antistress and Antioxidant Effects of Fermented and Germinated Mung Bean

**DOI:** 10.1155/2014/694842

**Published:** 2014-04-29

**Authors:** Swee Keong Yeap, Boon Kee Beh, Norlaily Mohd Ali, Hamidah Mohd Yusof, Wan Yong Ho, Soo Peng Koh, Noorjahan Banu Alitheen, Kamariah Long

**Affiliations:** ^1^Institute of Bioscience, Universiti Putra Malaysia, 43400 Serdang, Selangor, Malaysia; ^2^Department of Bioprocess Technology, Faculty of Biotechnology and Biomolecular Science, Universiti Putra Malaysia, 43400 Serdang, Selangor, Malaysia; ^3^Department of Cell and Molecular Biology, Faculty of Biotechnology and Biomolecular Science, Universiti Putra Malaysia, 43400 Serdang, Selangor, Malaysia; ^4^School of Biomedical Sciences, The University of Nottingham, Malaysia Campus, Jalan Broga, 43500 Semenyih, Selangor, Malaysia; ^5^Biotechnology Research Centre, Malaysian Agricultural Research and Development Institute (MARDI), 43400 Serdang, Selangor, Malaysia

## Abstract

Mung bean has been traditionally used to alleviate heat stress. This effect may be contributed by the presence of flavonoids and **γ**-aminobutyric acid (GABA). On the other hand, fermentation and germination have been practised to enhance the nutritional and antioxidant properties of certain food products. The main focus of current study was to compare the antistress effect of none-process, fermented and germinated mung bean extracts. Acute and chronic restraint stresses were observed to promote the elevation of serum biochemical markers including cholesterol, triglyceride, total protein, liver enzymes, and glucose. Chronic cold restraint stress was observed to increase theadrenal gland weight, brain 5-hydroxytryptamine (5-HT), and malondialdehyde (MDA) level while reducing brain antioxidant enzyme level. However, these parameters were found reverted in mice treated with diazepam, high concentration of fermented mung bean and high concentration of germinated mung bean. Moreover, enhanced level of antioxidant on the chronic stress mice was observed in fermented and germinated mung bean treated groups. In comparison between germinated and fermented mung bean, fermented mung bean always showed better antistress and antioxidant effects throughout this study.

## 1. Introduction

Stress response is a behaviour survival mechanism that helps fortify the physical and mental condition [1]. Neurotransmitters including noradrenaline (NA), dopamine (DA), and 5-hydroxytryptamine (5-HT) are the important biogenic monoamines. Under stress condition, NA and 5-HT will be highly elevated [2, 3]. However, extreme stress can cause impaired dopamine neurons [1] and collapse the homeostasis which may lead to various psychotic disorders including immunosuppression, hypertension, anxiety, endocrine disorder, and behaviour disorder [4]. Besides, restraints stress can be associated with the production of reactive oxygen species that may contribute to tissue damage [2]. Various drugs including diazepam, amphetamines, caffeine, and some anabolic steroids have been used and misused currently to overcome stress. The overdependence or misuse of these drugs often is associated with issues of toxicity and side effects [4].

Herbal medicines have been proposed as a cheaper and safer potential antistress agent. For examples,* Withania somnifera, Piper longum, Momordica charantia, *and* Asparagus racemosus *have been reported to have adaptogenic effect [2, 3, 5]. Legumes have also received great attention due to the presence of phytochemicals and their bioactivities. Mung bean (*Vigna radiata*) is a type of legume that is commonly consumed in Asian countries as a source of carbohydrate [6]. It has now been becoming another popular functional food for promoting good health. In Asian countries, mung bean beverages are often used to prevent or eliminate heatstroke. Flavonoids including vitexin and isovitexin have been proven as the major contributor to this protective effect against heat stress and to reduction of oxidative stress [7]. Other than its antioxidant property, mung bean also possessed anti-inflammatory [6, 8], antityrosinase [9], and antiproliferative [10] effects. Fermentation and germination have been proposed as processes that can improve the quality of cereal and legume seeds. For instance, functional nutrients including *γ*-aminobutyric acid (GABA) and antioxidant compounds were enhanced through germination [11, 12] and fermentation processes [13]. Although the protective effect against heat stress of mung bean has been reported, the effect of mung bean as potential antistress agent on restraint stress is yet unknown.

The purpose of this study was to compare the antistress activity of none-process, fermented and germinated mung bean extracts on acute and chronic restraint stress. Regulations of brain NA, DA, 5-HT, and antioxidant level under chronic stress condition were also examined.

## 2. Materials and Methods

### 2.1. Preparation of Fermented Mung Bean Extracts

Mung bean seeds were purchased in October 2010 from a local store in Serdang, Selangor. It was dehulled, washed, and soaked in chilled water at room temperature for 18 hours before being steamed for 40 minutes. Then, the steamed mung bean was fermented using* Rhizopus sp. *strain of 5351 inoculums under solid-state condition at 30°C for another 48 hours. The fermented mung bean was dried, ground into powder, and extracted with deionised water for 30 minutes at room temperature [13]. Finally, the water extract was freeze-dried (yield 25%, w/w) and subjected to GABA determination.

### 2.2. Detection of GABA Content in Fermented Mung Bean Extracts

GABA content of freeze-dried fermented mung bean was determined using Waters Acquity UPLC system with UV-PDA detector set at wavelength of 260 nm. Acquity UPLC AccQ Tag Ultra Column (2.1 mm i.d. × 100 mm × 1.7 *μ*m particle size) was used together with operating oven column temperature of 55°C and flow rate at 0.7 mL/min. Mobile phase consisted of AccQ Tag Ultra Eluent A (mobile phase A) and AccQ Tag Ultra Eluent B (mobile phase B) that were used under linear gradient condition as follows: 0.1% Eluent B under isocratic flow for 0.54 minutes and increased from 0.1% to 9.1% Eluent B for 5.20 minutes. Eluent B was then increased to 21.2% for another 2 minutes followed by another increment to 59.6% Eluent B for 1.06 minutes before reverting back to 0.1% Eluent B for 2.1 minutes. Finally, reconditioning the column with 0.1% Eluent B with isocratic flow for 0.30 min, data collected was analyzed using Waters Empower 2 software [14].

### 2.3. Animals

Male Balb/c mice (aged 8 weeks with average body weight of 25 ± 2 g) obtained from Animal Housing Department, Institute of Bioscience, Universiti Putra Malaysia, were used for all the tests below. Mice were kept in prebeded plastic cages under controlled conditions of 22 ± 1°C and standard 12 hours of dark/light day cycles with food and water* ad libitum*. Procedures for this study were carried out according to the guideline of National Institute of Health for the Care and Use of Laboratory Animals. This study was approved by the Animal Care and Use Committee, Universiti Putra Malaysia (UPM).

### 2.4. Acute Restraint Stress

Mice were randomly divided into 8 groups with 8 animals each. Group 1 (nonstress control) and group 2 (untreated control) were fed with 0.2% sodium carboxymethyl cellulose in saline, group 3 (positive control) received 2 mg/kg of diazepam, group 4 received 1000 mg/kg of none process mung bean extracts, groups 5 and 6 received 250 or 1000 mg/kg of fermented mung bean extracts, and groups 7 and 8 received 250 or 1000 mg/kg of germinated mung bean extracts. All treatments were given p.o. for 7 days. On the 7th day, mice were tied and immobilized with adhesive tape for 2 hours. At the end of the test, mice were sacrificed and blood was collected to obtain serum for determination of glucose, total cholesterol, triglyceride (TG), alkaline phosphatase (ALP), and alanine aminotransferase (ALT) (Biovision, USA) level according to the manufacturers' protocol [15].

### 2.5. Chronic Cold Restraint Stress

Mice were randomly divided into 8 groups with 8 animals each and the grouping was the same as the acute restraint stress test. All treatments were given p.o. for 21 days. After that, all mice except for group 1 (nonstress control) were exposed to cold restraint stress (placed under 4°C for 1 hour) continuously for another 7 days. Treatments were continued during this period. On the last day of the experiment, mice were treated, exposed to cold restraint stress, and sacrificed and blood was collected to obtain serum for determination of glucose, total cholesterol, triglyceride (TG) (Biovision, USA), and corticosterone (Cayman, USA) level according to the manufacturers' protocol. Moreover, adrenal gland was removed and weighted. Brains were frozen in liquid nitrogen and homogenized in mixture of HCl (0.01 N) and butanol. The aqueous phase acid extract was recovered by centrifugation and concentrations of dopamine (DA) (IBL, Hamburg, Germany) and 5-hydroxytryptamine (5-HT) (IBL, Hamburg, Germany) were quantified using ELISA [15] while brain superoxide dismutase (SOD) and malondialdehyde (MDA) levels were quantified according to the literature [16].

### 2.6. Statistical Analysis

Results from all the above tests were presented as mean ± standard deviation values. One-way analysis of variance (ANOVA) with post hoc Duncan test was used to evaluate statistical significance of the above results.* P*  values < 0.05 were considered as significant.

## 3. Results

### 3.1. GABA Content in Fermented and Germinated Mung Bean Extracts

The concentrations of GABA in fermented and germinated mung bean extracts were 0.131 ± 0.015 and 0.502 ± 0.035 g/100 g, respectively [17].

### 3.2. Acute Restraint Stress

Acute restraint stress significantly raised the level of serum cholesterol, TG, total protein, and glucose as compared to untreated normal control. The effects of fermented and germinated mung bean extracts were comparable to diazepam in the reduction of mice serum biochemical profiles under acute restraint stress condition (Table 1).

### 3.3. Chronic Restraint Stress

Chronic cold restraint stress has significantly increased the serum biochemical levels (cholesterol, TG, total protein, glucose, and corticosterone), adrenal gland weight, and brain MDA level. On the other hand, it also reduced brain antioxidant enzyme level (SOD), brain serotonin (5-HT), and spleen weight (Table 2). Fermented and germinated mung bean extracts (both at 1000 mg/kg concentration) and diazepam were able to restore these parameters back to nonstress normal control level. The antistress effects of fermented and germinated mung bean extracts were in a dosage dependent manner where low dosage of these extracts especially from germinated mung bean possessed the lowest antistress effect among all the tested samples. However, no statistical significance was observed in brain dopamine (DA) level of all the control and treated groups.

## 4. Discussion

Adaptation is a common central neurotransmission response toward stress and this event can help strengthen the organisms to handle stress [2]. Under stress condition, various adrenal hormones are released and these hormones will induce insulin resistance which results in the elevation of plasma glucose level. Furthermore, the release of NA and corticosteroid will also stimulate hyperinsulinemia and thus raise the synthesis of cholesterol. These changes affect the mobilisation of stored fat and carbohydrate reserves that eventually raise the level of blood cholesterol, TG, total protein, and glucose [15, 18]. Thus, the depletion of monoamines (NA and DA) was often associated with high level of serum biochemical profiles under overstress condition. According to American Academy of Family Physicians, stress related problems including insomnia and depression have contributed to large percentage of cases in primary health care [19]. Various antistress and antidepressant drugs have been developed; however, the associations of toxicity and side effects issues have limited their therapeutic practice [4]. Thus, food base natural products may be a better and more convenient alternative to assist in stress management in primary health care.

Mung bean was found to be an excellent dietary source of natural antioxidants for health promotion [10] and this antioxidant effect has contributed to its antiheat stress activity [7]. In this study, we evaluated the antistress effect of fermented and germinated mung bean extracts toward acute restraint stress and chronic cold restraint stress. Diazepam, a nonspecific antistress agent [15], was used as positive control in this study. Overall reduction of cholesterol, TG, total protein, and glucose levels may be contributed by the reduction of corticosteroid level in the fermented mung bean extracts treated group. Fermented food such as fermented rice bran has been reported as antistress and antifatigue agent [20]. Our results showed that fermented and germinated mung bean extracts possessed better antistress effect in a dosage dependent manner for both acute and chronic stress model than none-process mung bean extracts. This effect may be contributed by the presence of GABA in both fermented and germinated mung bean extracts. GABA is a nonprotein amino acid that works as an inhibitor to neurotransmission [21]. It plays an important role in central integration of hypothalamic-pituitary-adrenocortical (HPA) stress reaction. None adequate level of GABA in plasma has been related to the development of acute posttraumatic stress disorder [21, 22]. On the other hand, previous study has reported that ethanol extract of* Rubia cordifolia *can stimulate the brain GABA level that subsequently reduced the dopamine and plasma corticosterone levels [18]. The GABA contents of our fermented and germinated mung bean (0.131 ± 0.015 and 0.502 ± 0.035 g/100 g, resp.) were recorded to be higher than those detected in germinated brown rice (0.0818 ± 0.0072 g/100 g). This may be contributed by the different level of GABA-synthesizing enzyme that is present in different plants [23]. In this study, germinated mung bean extracts contained higher level of GABA as compared to fermented mung bean extracts. However, fermented mung bean extracts gave the best antistress effect in all the experiments in a dosage dependent manner while germinated mung bean extracts only possessed slightly better effect as compared to none-process mung bean extracts. This phenomenon may be contributed by the strong antioxidant activity found in fermented mung bean extracts which concurrently protected the damage induced by stress [17]. Stress also promotes the development of reactive oxygen species and free radicals that lead to the formation of lipid peroxides. Nervous system is very sensitive to peroxidative damage during restraints stress due to the increase of oxygen tension and reduction of antioxidant level [24]. Thus, effective antioxidants that can inhibit lipid oxidation may help prevent organ damage during stress conditions. Previous study has shown that polyphenol from oolong tea was able to reduce the stress and the stress-induced lipid peroxide levels on women loaded with vigil [25]. To correlate the antistress capability with the antioxidant effects of fermented mung bean extracts, the levels of antioxidant SOD enzyme and lipid peroxidation in the brain were determined. In this study, we found that mice under chronic cold restraint stress contained high level of MDA with depletion of SOD level (Table 2). The decreased SOD level was significantly raised by fermented mung bean extracts in a dosage dependent manner. This effect was associated with mark reduction of MDA level.

High incidence of stress related mental illness has been recorded in primary health care nowadays. Healthy functional food is a more convenient and better choice in stress management. This study has demonstrated that fermented and germinated mung bean extracts possessed antistress activity in both acute and chronic stress mice. It also reverts the antioxidant level in brain under stress condition. Thus, fermented mung bean extracts exhibited great potential to be developed into functional foods or nutraceutical ingredients for reducing oxidative stress and as alternative antistress agent for primary mental health problem.

## Figures and Tables

**Table 1:** Effect of fermented and nonfermented mung bean extracts on serum biochemical profile under acute restraint stress.

	Chol (mmol/L)	Trig (mmol/L)	Total protein (g/dL)	Glucose (mmol/L)
Untreated normal control	6.23 ± 0.27*	3.96 ± 0.78*	138.78 ± 6.70*	6.93 ± 0.51*
Untreated stress control	8.32 ± 0.87	5.43 ± 0.30	157.88 ± 13.15	7.85 ± 0.07
Diazepam (2 mg/kg)	5.32 ± 0.86*	2.55 ± 0.49*	104.78 ± 25.03*	5.40 ± 0.26*
None-process mung bean (1000 mg/kg)	7.21 ± 0.77*	3.96 ± 0.35*	147.24 ± 4.14	6.14 ± 1.66*
Fermented mung bean (250 mg/kg)	6.58 ± 0.66*	4.18 ± 0.65*	142.54 ± 1.06	6.07 ± 0.92*
Fermented mung bean (1000 mg/kg)	5.27 ± 0.92*	3.14 ± 0.81*	108.48 ± 10.82*	5.47 ± 0.35*
Germinated mung bean (250 mg/kg)	8.73 ± 0.85	5.86 ± 0.33	155.43 ± 11.16	7.51 ± 0.13
Germinated mung bean (1000 mg/kg)	5.54 ± 0.74	2.34 ± 0.62	100.02 ± 21.63	5.59 ± 0.38

*P > 0.05 versus untreated stress control for acute restraint test.

**Table 2:** Effect of fermented and nonfermented mung bean extracts on serum biochemical profile, adrenal gland weight, brain 5-hydroxytryptamine (5-HT), superoxide dismutase (SOD), and malondialdehyde (MDA) levels under chronic cold restraint stress.

	Chol (mmol/L)	Trig (mmol/L)	Total protein (g/dL)	Glucose (mmol/L)	Corticosterone (µg/100 mL)	Adrenal gland weight (mg/100 g)	Brain 5-HT level (µg/g)	Brain SOD (U/mg protein)	Brain MDA (nmol/g of protein)
Untreated normal control	5.11 ± 0.56*	2.82 ± 0.30*	97.90 ± 8.10	5.32 ± 0.71*	8.58 ± 0.75*	31.07 ± 2.43*	0.08 ± 0.07*	11.35 ± 1.22*	2.37 ± 0.71*
Untreated stress control	5.87 ± 0.54	3.19 ± 0.88	118.37 ± 22.04	9.60 ± 0.14	14.74 ± 0.35	35.13 ± 3.09	2.20 ± 0.07	4.58 ± 1.83	15.63 ± 1.44
Diazepam (2 mg/kg)	4.06 ± 0.90*	1.70 ± 0.49*	92.43 ± 12.42*	5.07 ± 0.49*	8.81 ± 0.49*	30.62 ± 3.33*	0.73 ± 0.09*	8.32 ± 1.67*	8.49 ± 1.51*
None-process mung bean (1000 mg/kg)	4.83 ± 0.11*	3.62 ± 0.36	99.52 ± 6.47	9.04 ± 0.45*	8.90 ± 0.14*	27.53 ± 2.12*	1.72 ± 0.05*	6.33 ± 1.25*	11.57 ± 1.11*
Fermented mung bean (250 mg/kg)	5.15 ± 0.16*	2.98 ± 0.19	97.00 ± 2.83*	9.40 ± 1.56	9.52 ± 0.51*	30.05 ± 2.75*	2.10 ± 0.09	7.16 ± 1.15*	9.64 ± 2.10*
Fermented mung bean (1000 mg/kg)	4.19 ± 0.08*	3.16 ± 0.70	91.05 ± 7.71*	8.13 ± 0.99*	7.43 ± 0.31*	26.06 ± 1.50*	1.20 ± 0.03*	9.14 ± 2.36*	7.32 ± 1.86*
Germinated mung bean (250 mg/kg)	4.66 ± 0.70*	3.20 ± 0.65*	98.30 ± 6.53*	10.00 ± 1.05	15.01 ± 0.64	36.70 ± 1.50	1.70 ± 0.06*	5.79 ± 1.28	10.46 ± 0.36*
Germinated mung bean (1000 mg/kg)	4.77 ± 0.13*	3.33 ± 0.99*	96.17 ± 10.61*	9.04 ± 0.61*	8.90 ± 0.14*	26.97 ± 1.49*	1.65 ± 0.06*	7.81 ± 1.47*	10.17 ± 0.49*

*P > 0.05 versus untreated stress control for chronic cold restraint test.

## References

[1] Pani L, Porcella A, Gessa GL (2000). The role of stress in the pathophysiology of the dopaminergic system. *Molecular Psychiatry*.

[2] Joshi T, Sah SP, Singh A (2012). Antistress activity of ethanolic of Asparagus racemosus Willd roots in mice. *Indian Journal of Experimental Biology*.

[3] Kavitha CN, Babu SM, Rao MEB (2011). Influence of Momordica charantia on oxidative stress-induced perturbations in brain monoamines and plasma corticosterone in albino rats. *Indian Journal of Pharmacology*.

[4] Desai SK, Desai SM, Navdeep S, Arya P, Pooja T (2011). Antistress activity of *Boerhaavia diffusa* root extract and a polyherbal formulation containing *Boerhaavia diffusa* using cold restraint stress model. *International Journal of Pharmacy and Pharmaceutical Sciences*.

[5] Khan MN, Suresh J, Yadav KSH, Ahuja J (2012). Formulation and evaluation of antistress polyherbal capsule. *Der Pharmacia Sinica*.

[6] Lee S-J, Lee JH, Lee H-H (2011). Effect of mung bean ethanol extract on pro-inflammtory cytokines in LPS stimulated macrophages. *Food Science and Biotechnology*.

[7] Cao D, Li H, Yi J (2011). Antioxidant properties of the mung bean flavonoids on alleviating heat stress. *PLoS ONE*.

[8] Ismail GSM (2012). Protective role of nitric oxide against arsenic-induced damages in germinating mung bean seeds. *Acta Physiologiae Plantarum*.

[9] Yao Y, Cheng X, Wang L, Wang S, Ren G (2012). Mushroom tyrosinase inhibitors from mung bean (*Vigna radiatae* L.) extracts. *International Journal of Food Sciences and Nutrition*.

[10] Xu B, Chang SKC (2012). Comparative study on antiproliferation properties and cellular antioxidant activities of commonly consumed food legumes against nine human cancer cell lines. *Food Chemistry*.

[11] Fernandez-Orozco R, Frias J, Zielinski H, Piskula MK, Kozlowska H, Vidal-Valverde C (2008). Kinetic study of the antioxidant compounds and antioxidant capacity during germination of *Vigna radiata* cv. emmerald, Glycine max cv. jutro and Glycine max cv. merit. *Food Chemistry*.

[12] Sutharut J, Sudarat J (2012). Total anthocyanin content and antioxidant activity of germinated colored rice. *International Food Research Journal*.

[13] Tsai JS, Lin YS, Pan BS, Chen TJ (2006). Antihypertensive peptides and *γ*-aminobutyric acid from prozyme 6 facilitated lactic acid bacteria fermentation of soymilk. *Process Biochemistry*.

[14] Guo Y, Chen H, Song Y, Gu Z (2011). Effects of soaking and aeration treatment on *γ*-aminobutyric acid accumulation in germinated soybean (*Glycine max* L.). *European Food Research and Technology*.

[15] Kulkarni M, Juvekar A (2008). Attenuation of acute and chronic restraint stress-induced perturbations in experimental animals by *Nelumbo nucifera* Gaertn. *Indian Journal of Pharmaceutical Sciences*.

[16] Ho WY, Liang WS, Yeap SK, Beh BK, Yousr AHN, Alitheen NB (2012). *In vitro* and *in vivo* antioxidant activity of *Vernonia amygdalina* water extract. *African Journal of Biotechnology*.

[17] Ali NM, Yusof HM, Long K (2013). Antioxidant and hepatoprotective effect of aqueous extract of germinated and fermented mung bean on ethanol-mediated liver damage. *BioMed Research International*.

[18] Kenjale RD, Shah RK, Sathaye SS (2007). Anti-stress and anti-oxidant effects of roots of chlorophytum borivilianum santa pau & fernandes. *Indian Journal of Experimental Biology*.

[19] American Academy of Family Physicians Mental health care services by family physicians (Position paper). http://www.aafp.org/online/en/home/policy/policies/m/mentalhealthcareservices.html.

[20] Kim KM, Yu KW, Kang DH, Koh JH, Hong BS, Suh HJ (2001). Anti-stress and anti-fatigue effects of fermented rice bran. *Bioscience, Biotechnology and Biochemistry*.

[21] Vaiva G, Thomas P, Ducrocq F (2004). Low posttrauma GABA plasma levels as a predictive factor in the development of acute posttraumatic stress disorder. *Biological Psychiatry*.

[22] Patel NB, Galani VJ, Patel BG (2011). Antistress activity of *Argyreia speciosa* roots in experimental animals. *Journal of Ayurveda and Integrative Medicine*.

[23] Thitinunsomboon S, Keeratipibul S, Boonsiriwit A (2013). Enhancing gamma-aminobutyric acid content in germinated brown rice by repeated treatment of soaking and incubation. *Food Science and Technology International*.

[24] Metodiewa D, Koska C (2000). Reactive oxygen species and reactive nitrogen species: relevance to cyto (neuro) toxic events and neurologic disorders. An overview. *Neurotoxicity Research*.

[25] Kurihara H, Chen L, Zhu B-F (2003). Anti-stress effect of oolong tea in women loaded with vigil. *Journal of Health Science*.

